# ROS1 amplification mediates resistance to gefitinib in glioblastoma cells

**DOI:** 10.18632/oncotarget.3981

**Published:** 2015-05-04

**Authors:** Hashim Aljohani, Robert F. Koncar, Ahmad Zarzour, Byung Sun Park, So Ha Lee, El Mustapha Bahassi

**Affiliations:** ^1^ Department of Internal Medicine; Division of Hematology and Oncology, Cincinnati, OH, USA; ^2^ Department of Internal Medicine, Cleveland Clinic Foundation, Cleveland, OH, USA; ^3^ Chemical Kinomics Research Center, Korea Institute of Science and Technology, Hwarangno, Seongbuk-gu, Seoul, Republic of Korea

**Keywords:** tyrosine kinase inhibitors, EGFR, gefitinib, ROS1, DDR1

## Abstract

Glioblastoma (GBM) is the most aggressive brain tumor in adults and remains incurable despite multimodal intensive treatment regimens. The majority of GBM tumors show a mutated or overexpressed EGFR, however, tumors treated with tyrosine kinase inhibitors (TKIs) will inevitably recur highlighting the need to identify signalling pathways involved in GBM resistance to these drugs. To this end, we treated GBM cells that overexpress EGFR with increasing concentrations of gefitinib and isolated resistant clones. These resistant clones were subject to RNAseq and the expression of several genes was found to be upregulated. These genes are mainly tyrosine kinase receptors and include ROS1, DDR1 and PDGFRA and are known to control several downstream targets of EGFR. The upregulation of ROS1 and DDR1 was confirmed at the protein level by western blot. Treatment with a potent and highly specific pyrazole ROS1 inhibitor in ROS1 overexpressing clones led to a sensitization of these cells to low concentrations of gefitinib. Combined treatment with gefitinib and ROS1 inhibitor induces massive cell death by apoptosis following a prolonged S phase cell cycle arrest. Our current study led to the discovery of alternative pathways used by GBM cells to evade cell death following treatment with gefitinib and identifies new therapeutic targets to prevent GBM cell resistance to the drug.

## INTRODUCTION

Glioblastoma (GBM) is the most common primary malignant brain tumor in adults [1, 2]. Despite multimodal therapy with radiation and the alkylating agent temozolomide, median survival is a dismal 15 months [3]. The most common genetic aberration associated with GBM is amplification of the epidermal growth factor receptor gene (*EGFR*, also referred to as *ERBB1* or *HER1*), with a frequency of about 50% [1]. EGFR is a member of the HER superfamily of receptor tyrosine kinases, together with ERBB2, ERBB3, and ERBB4 [4]. The structure of each of the members comprises: a ligand-binding ectodomain with 2 cysteine-rich regions; a single transmembrane region; and a cytoplasmic tyrosine kinase (TK) domain [5]. Binding of a cognate ligand to the ligand-binding site results in the autophosphorylation of the receptor and induction of downstream signaling through the PI3K/Akt and the MAPK pathways, among others [4] leading to cell differentiation, proliferation, and survival [6].

*EGFR* amplification and mutations are also found in breast, lung, and prostate cancers [7]. In spite of this, therapies that have been effective for these solid tumors have shown limited efficacy against GBM. EGFR-specific inhibitors have been approved for use in patients with non-small cell lung carcinoma (NSCLC), and are currently in clinical trials for GBM [8-10]. However, the clinical experience has been that many GBM patients do not respond to these therapies and those that do eventually show progression [11]. Successful treatment of GBM continues to be a major therapeutic challenge due to both inherent and acquired resistance [12, 13]. Mechanisms causing resistance to EGFR inhibitors have been studied in a number of solid tumors. Some of the documented mechanisms include the acquisition of secondary *EGFR* point mutations, co-activation and/or amplification of other receptor tyrosine kinases (RTKs), and up-regulation of drug efflux pumps, however, mechanisms of resistance that are unique to glioma are not clearly defined [12, 13].

Specific drugs that target EGFR signaling include erlotinib and gefitinib, which reversibly inhibit the EGFR tyrosine kinase domain by competitively binding with ATP, and the monoclonal antibodies (mAbs) cetuximab (a chimeric mouse-human IgG1 antibody) and panitumumab (a fully humanized IgG2 antibody). Cetuximab and panitumumab block ligand binding to the extracellular domain of EGFR, promote receptor internalization and mediate antibody- and complement-mediated cytotoxicity [14]. The common *EGFR*-activating mutations, exon 19 deletions and L858R, which account for 85% of all *EGFR* mutations, predict sensitivity to the EGFR TKIs (gefitinib, erlotinib and afatinib) in preclinical models and in patients with lung cancer. However, these mutations are largely absent in brain tumors.

To determine the mechanism by which glioblastoma cells acquire resistance to RTK inhibitors, U87 cells overexpressing EGFR were treated with increasing concentrations of gefitinib and resistant clones were isolated, expanded and subject to RNA sequencing (RNAseq). Data analysis revealed that the resistant clones show overexpression of the orphan RTK c-ros oncogene 1 (ROS1), discoidin domain receptor tyrosine kinase 1 (DDR1) or the platelet-derived growth factor receptor, alpha (PDGFRA). Other proteins from the AKT/mTOR pathway were also mildly amplified. Overexpression of ROS1 and DDR1 proteins was confirmed by western blotting. Using a pyrazole ROS1 inhibitor in four of the resistant clones, we were able to sensitize them to gefitinib confirming that the resistance was mediated by ROS1 in these cells. We also showed that both gefitinib and ROS1 inhibitors induce cell death by apoptosis following an S phase cell cycle arrest.

## RESULTS

### Identification of ROS1 and DDR1 as mediators of gefitinib resistance in U87 cells overexpressing EGFR protein

**Figure 1:**
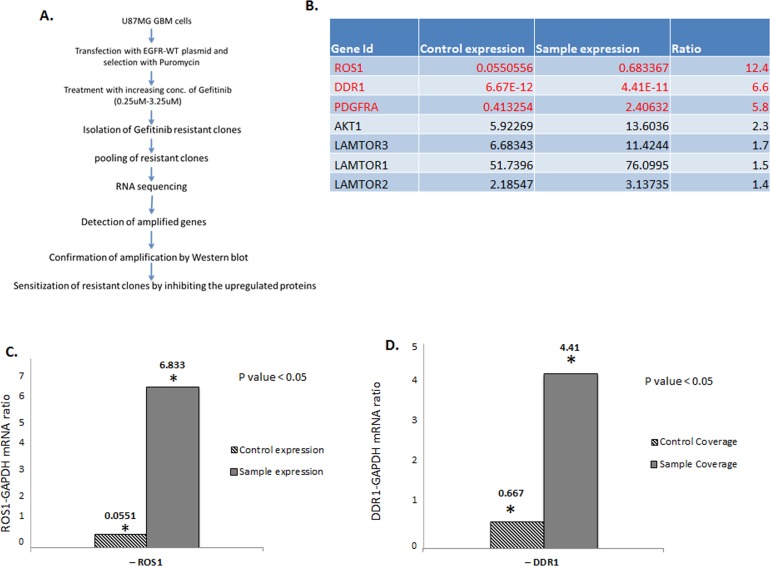
ROS1 and DDR1 mRNA is upregulated in gefitinib-resistant GBM cells **A.** Outline of the experimental strategy used to isolate and characterize the gefitinib-resistant cells. **B.** A list of the most upregulated genes in the gefitinib-resistant clones. **C.** A ratio of ROS1 and GAPDH1 control and **D.** a ratio of DDR1 and GAPDH control.

To identify genes and pathways that mediate resistance to the EGFR inhibitor gefitinib, U87 glioma cells expressing high levels of EGFR (U87-EGFR) were treated with increasing concentrations of the drug. Kill curve assay showed that the gefitinib IC_50_ concentration for U87-EGFR is 0.75 μM. We therefore started the screen at 0.75 μM and gradually escalated the dose up to 3.25 μM over a period of eight weeks. Cells that survived at this concentration were expanded, pooled together, and subject to RNA-seq. Non treated U87-EGFR gefitinib-sensitive cells were used as controls. The study design is described in Figure 1A. Three plates from either non treated or treated cells were used for RNA extraction and RNA sequencing. RNA-seq results show that besides a statistically significant increase in AKT1, AKT2, AKT3, PDGFB, LAMTOR1, LAMTOR2, LAMTOR3 and FIGF (Figure 1B), three tyrosine kinase receptor genes ROS1, DDR1 and PRGFRA showed the most significant increase in the gefitinib resistant cells. Figure 1B-1D shows a 12 times increase in ROS1 transcript in gefitinib-resistant cells compared to non-treated cells. Similarly, DDR1 transcript levels were much higher in the gefitinib-resistant cells compared to the sensitive ones (Figure 1D). This increase was not specific to U87-EGFR cells and was also observed in a low passage tumor-derived GBM cell line (Figure S1). While PDGFRA overexpression has been show to mediate resistance to EGFR inhibitors, to our knowledge, this is the first report of the involvement of ROS1 and DDR1 in TKI resistance in GBM. Interestingly, a survey of The Cancer Genome Atlas (TCGA) data indicates that ROS1 and DDR1 upregulation correlates with shorter overall survival (OS) and progression free survival (PFS) (Figure S2).

### The increase in ROS1 and DDR1 transcripts in the resistant cells correlates with an increase in protein expression

**Figure 2:**
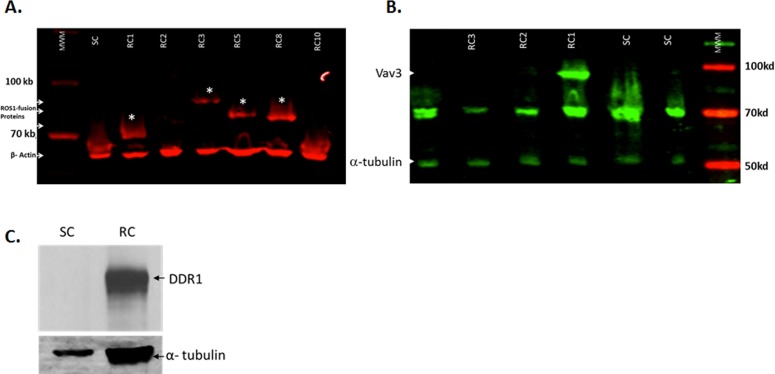
ROS1 and DDR1 protein expression in gefitinib-resistant cells To confirm that the increase in mRNA expression translates to an increase in protein expression, several clones were tested for the expression of ROS1 protein. **A.** Four resistant clones (RC) show the expression of different ROS1 fusion proteins (indicated by asterisk). The sensitive clone control (SC) did not show such fusions. **B.** Resistant clone #1 shows an overexpression of Vav 3, a target of ROS1 and a potential ROS1 ligand as well. **C.** The gefitinib-resistant cell pool was also tested for the upregulated expression of the DDR1 protein.

Oncogenic activation of ROS1 is observed in a subset of patients with glioblastoma, non–small-cell lung cancer (NSCLC), and cholangiocarcinoma [15-17]. In most cases, ROS1 signaling is activated by interchromosomal translocation or intrachromosomal deletion that results in N-terminal *ROS1* fusion genes. Several ROS1 kinase fusion proteins have been identified, including the Fused in Glioblastoma–ROS1 (FIG–ROS) that was first discovered in a human glioblastoma cell line [18] and more recently in patients with NSCLC [19], cholangiocarcinoma [17], and serous ovarian carcinoma [18]. The SLC34A2*–*ROS1 (SLC–ROS) fusion is present in a subset of patients with NSCLC [13, 19] and gastric cancer [20]. Other ROS1 fusions include CD74*–*ROS1, EZR*–*ROS1, LRIG3*–*ROS1, SDC4*–*ROS1, and TPM3*–*ROS1 [17]. To test whether the observed increase in transcript levels of ROS1 and DDR1 translates to an increase in protein level expression, lysates from gefitinib-resistant and gefitinib-senstive cells were tested for ROS1 protein expression by western blotting. To this end, single clones were derived from the original pool of resistant cells. Single clones were expanded and tested by western blotting for the expression of ROS1 protein. Four clones showed overexpression of different ROS1 fusion proteins (Figure 2A). Interestingly, one of the four clones (RC1) shows both overexpression of ROS1 and its ligand VAV3 (Figure 2B), a Rho GTPase guanine nucleotide exchange factor, associated with tumor growth, apoptosis, invasion and metastasis, and angiogenesis, which has also been shown to be phosphorylated and activated by ROS1 [21]. The pooled resistant cells were also tested for DDR1 protein expression. DDR1 is a receptor tyrosine kinase that is identified during the search for tyrosine kinase proteins expressed in human malignancies [22]. DDR1 kinase contains a homology domain to discoidin, which is distinct from other members of the large receptor tyrosine kinase and could be activated by various types of collagens and is found to be involved in cell attachment, migration and invasion [23]. Accumulated evidence indicates that DDR1 is overexpressed in invasive tumors including breast, prostate, lung and cancer cells overexpressing DDR1 display increased migration and invasion [24, 25]. For instance, upregulated DDR1 expression promotes cancer development by enhancing cancer cell survival and invasion, and high DDR1 expression is associated with short hormone resistance interval in prostate carcinoma [26]. Figure 2C shows that while the sensitive cells show an almost undetectable level of endogenous DDR1 protein, DDR1 protein is highly expressed in lysates from the resistant cells. These data show that ROS1 and DDR1 are upregulated both at the RNA and protein levels and may be responsible for resistance to gefitinib. Since ROS1 inhibitors are readily available, we decided to follow up on the ROS1 overexpressing clones and test the possibility of rendering them sensitive to low concentrations of gefitinib using a pyrazole ROS1 inhibitor that we have previously shown to specifically target ROS1 protein but not a large panel of kinases including tyrosine kinase receptor proteins.

### Gefitinib-resistant cells that overexpress ROS1 protein are highly sensitive to ROS1 inhibitors

**Figure 3:**
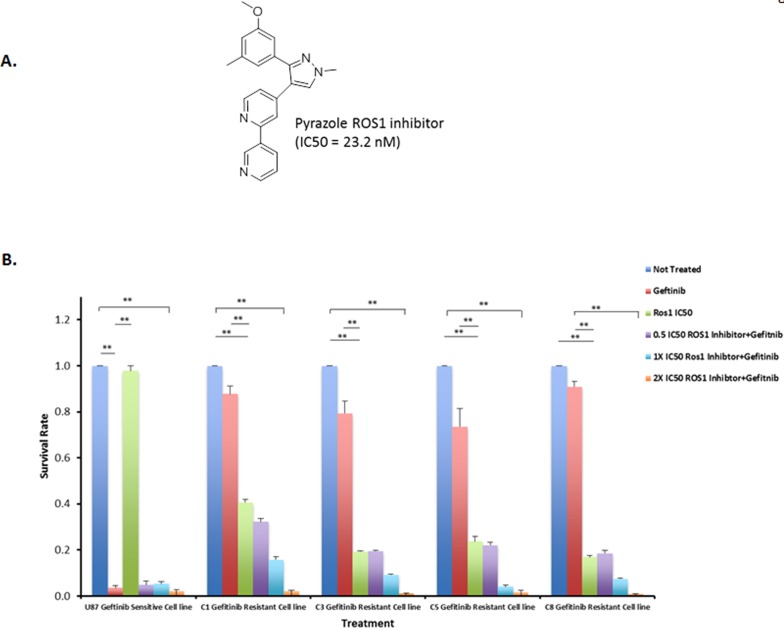
ROS1 inhibitor sensitizes gefitinib-resistant cells to the gefitinib **A.** Structure of pyrazole ROS1 inhibitor. **B.** Gefitinib and ROS1 Inhibitor combined treatment of U87-EGFR clones that are either sensitive or resistant to gefetinib at 36 hrs post-treatment. C1, C3, C5 and C8 indicate the four resistant clones that overexpress ROS1 fusions.

We have previously described a number of pyrazole compounds that have a potent and selective activity with IC_50_ value of 199 nM for ROS1 [27]. Recently, we synthesized a new pyrazole compound that shows excellent inhibition of ROS1 enzymatic activity with an IC_50_ value of 23.2 nM and it also showed high selectivity for ROS1 kinase (Figure 3A) [28]. To test whether gefitinib-resistant cells that overexpress ROS1 fusions can be sensitized with the ROS1 inhibitor to low concentrations of gefitinib, the four clones that show over-expression of the ROS1 fusions as well as gefitinib-sensitive cells were subject to increasing concentrations of the ROS1 inhibitor either singly or in combination with 1 μM gefitinib. Data in Figure 3B show that gefitinib-resistant cells are sensitive to ROS1 inhibitor and a 2X the IC_50_ of the drug (23.2 nM) combined with 1 μM of gefitinib achieved an almost complete growth inhibition of the resistant cells in 36 hours while ROS1 inhibitor only did not show a dramatic effect on gefitinib-sensitive cells. Crizotinib, a FDA approved drug for treatment of Non-Small Cell Lung Carcinoma (NSCLC) patients with rearranged ROS1 was used to treat these resistant cells and showed a moderate effect compared to the pyrazole ROS1 inhibitor (Figure S3). These data show that ROS1 inhibition provide a therapeutic alternative in gefitinib-resistant cells that overexpress ROS1 fusions.

### ROS1 inhibition leads to S phase cell cycle arrest followed by cell death in gefitinib-resistant cells

**Figure 4:**
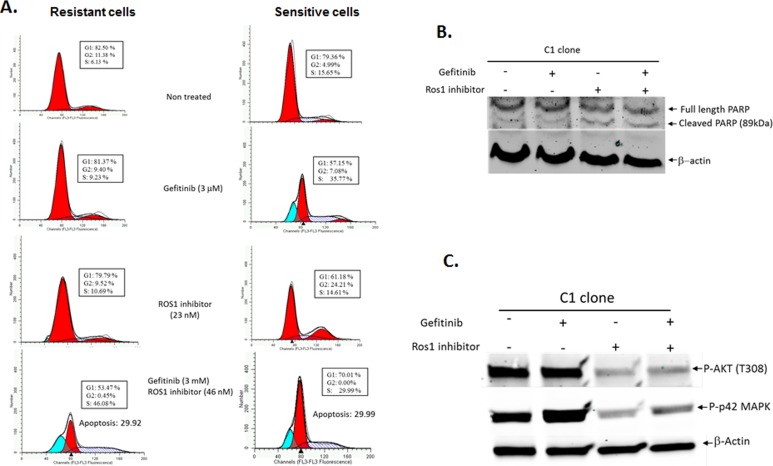
Cell cycle profile of gefitinib-sensitive and gefitinib-resistant glioblastoma cell lines following treatment with gefitinib, pyrazole ROS1 inhibitor or a combination of both **A.** Pyrazole ROS 1 inhibitor sensitizes gefitinib-resistant cells to gefitinib through a prolonged S phase checkpoint arrest followed by cell death by apoptosis as indicated by the accumulation of cells in sub-G1 phase of the cell cycle. **B.** Increased PARP cleavage in cells treated with ROS1 inhibitor and gefitinib. **C.** Inhibition of pAKT1 and p-p42MAPK proteins following treatment with gefitinib and ROS1 inhibitor.

To investigate the mechanism by which ROS1 inhibitor induces cell death in gefitinib-resistant cells, we analyzed the cell cycle profile of both gefitinib-sensitive and gefitinib-resistant cells after treatment with either gefitinib, ROS1 inhibitor or a combination of both inhibitors (Figure 4A). While the non-treated cells show a normal cell cycle distribution in both gefitinib-sensitive and gefitinib-resistant cells, despite small differences mainly in G2 and S phase populations, the cell cycle profile of gefitinib-resistant cells did not show any noticeable changes after treatment with 3 μM gefitinib, however the gefitinib-sensitive cells showed an increase in S phase population, a slight increase in G2 population and the accumulation of a sub-G1 population, suggestive of an active cell death likely by apoptosis. Treatment of gefitinib-resistant cells with gefitinib only led to a slight increase in S phase population. Treatment with pyrazole ROS1 inhibitor led to a slight increase S phase cells and the start of the appearance of a small sub-G1 population. Such a sub-G1 population was completely absent in the sensitive cells suggesting that sensitive cells do not rely on ROS1 for their growth. Combination of gefitinib and pyrazole ROS1 inhibitors led to a sharp S arrest, an almost complete absence of mitotic cells, a sharp decrease in G1 population and accumulation of cells in sub-G1 in both resistant and sensitive cells. These data indicate that ROS1 inhibition of cell growth in gefitinib-resistant cells is mediated through a prolonged S phase checkpoint arrest followed by accumulation of a sub-G1 population indicative of cell death by apoptosis. The apoptotic phenotype is supported by an increase in PARP cleavage in cells treated by the ROS1 inhibitor and ROS1 inhibitor combined with gefitinib (Figure 4B). Since ROS1 activates some of the same pathways that are activated by EGFR, we investigated whether the ROS1 inhibitor compound was able to inhibit the downstream EGFR effectors mainly AKT1 and p42 MAPK proteins. Indeed, while gefitinib alone was unable to inhibit the active forms of these proteins in the resistant clones, ROS1 inhibitor alone or in combination with gefitinib efficiently inhibited the expression of both p-AKT1 and p-p42 MAPK (Figure 4C).

## DISCUSSION

Resistance to EGFR therapy is an endemic problem faced in the clinic every day. While the majority of brain tumors show an overexpressed or mutant EGFR, TKI remain inefficient and even patients that show some response eventually recur as a result of acquired resistance. While in lung cancer, mutations that are associated with TKI sensitivity are well established and secondary mutations that lead to acquired resistance are also well known, no such mutations have been reported in brain tumors. To identify genetic determinants associated with resistance to EGFR therapy, and specifically to the TKI inhibitor, gefitinib, we carried out an RNA-seq of gefitinib-resistant and gefitinib-sensitive clones and identified three proteins that were highly expressed as a result of the inhibition of the EGRF receptor namely ROS1, DDR1 and PDGFRA. Of importance, all the three proteins are tyrosine kinase receptors themselves and regulate the same signaling pathways that are associated with EGFR activation. While PDFRA upregulation has been associated with TKI resistance, to our knowledge, there are no reports of ROS1 and DDR1 involvement in this process. We therefore followed up on DDR1 and ROS1 and confirmed that these proteins were upregulated at the protein level as well and not only at the RNA level. Blotting for ROS 1 protein also showed that several fusion proteins involving ROS1 were expressed. As indicated earlier, ROS1 is activated as a fusion protein with several proteins leading to a constitutively active chimeric protein. To further investigate the role of ROS1 overexpression in gefitinib resistance, we used the pyrazole ROS1 inhibitor that is very highly potent and specific and showed that this inhibitor sensitizes gefitinib-resistant cells to the drug at low concentrations. The mechanism by which ROS1 induces cell death in combination with gefitinib in both resistant and sensitive cells is through a cell cycle arrest in S phase and a subsequent cell death by apoptosis.

Given the recent success of molecularly targeted therapies in treating cancers driven by oncogenic kinases, there is acute clinical momentum to identify inhibitors that selectively target ROS1 fusions. Because the ROS1 and Anaplastic Lymphoma Kinase (ALK) domains are partially homologous, the Food and Drug Administration (FDA)-approved ALK/MET kinase inhibitor crizotinib is being investigated via phase I/II clinical trials for its efficacy in ROS1-driven lung cancer patients [29]. The ROS1 inhibitor described in this study showed a stronger inhibitory effect on ROS1 rearrangements compared to crizotinib. More recently, foretinib (GSK1363089) and Gö6976 were also shown to be potent inhibitors of ROS1 [30]. Foretinib was shown to selectively suppress the growth of ROS1 fusions-driven cell lines as well as of FIG–ROS-driven tumors in mice [30].

These data indicate that a genetic screen of tumors that develop resistance to TKI can reveal alternative pathways that drive resistance to the drugs and these pathways can be targeted to achieve cell death. Developing inhibitors to the proteins identified during this study will provide alternatives to patients with recurrent brain tumors that are refractory to EGFR therapy.

## MATERIALS AND METHODS

### Isolation of gefitinib-resistant clones

To isolate gefitinib-resistant clones, U87 GBM cells were transfected with a retroviral construct that expresses EGFR. Stable clones were isolated by selection with puromycin. EGFR Protein overexpression was confirmed by western blot. To isolate gefitinib-resistant clones, 70-80% confluent cells were treated with increasing concentrations of gefitinib starting from 0.75 μM/ml for 2 weeks. The remaining viable cells were then treated with 1 μM/ml of gefitinib, and repeated the procedures until we reached 3 μM/ml. Cells that were able to resist a 3 μM/ml were pooled together and used for RNA extraction and RNA sequencing.

### RNA Isolation and RNA sequencing

RNA was isolated from six different clones of U87 cells, three of which are resistant to 3 μM/ml of gefitinib and three are sensitive. These cells were grown as monolayer in DMEM media and lysed in the culture dish by addition of RNAzol®RT reagent (Molecular Research Center, Inc. Catalog No: RN 190). Culture medium was first removed and 1 ml of RNAzol®RT reagent was added per 3.5 cm culture dish (10 cm^2^). The lysate was then passed through a pipette several times and the RNA extracted according to the manufacturer's protocol. RNA was solubilized in RNAase free water by vortexing 2-5 min at room temperature to attain a typical yield of 1 to 2 μg/ml total RNA.

### RNA sequencing

RNA-seq was performed at the Cincinnati Children's Hospital Medical Center (CCHMC) Genetic Variation and Gene Discovery Core. In short, a library of cDNA fragments is formed from the extracted RNAs, and then sequencing adaptors were added to the cDNA library. Several short fragment sequences were sequenced and the sequencing reads were aligned to the reference transcriptome.

### MTS assay

To evaluate the proliferation and survival, we used CellTiter 96® AQueous one solution cell proliferation assay from Promega that measures the metabolic activity of the cell lines under each treatment condition, according to the standard protocol (Promega).

### Western blots

In order to confirm the protein expression of ROS1, DDR1 and VAV3, We used western blotting following standard protocols. A combination of chemiluminescent detection and LI-COR detection was used in this study. For the detection of ROS1 protein expression, we used ROS1 (69D6, Cell Signaling Technology Inc. catalogue #3266) mouse mAb. For detection of Vav3 protein expression, we used Vav3 antibody, which recognizes endogenous Vav3 protein, (Cell Signaling Technology Inc. catalogue #2398). For DDR1 protein detection, we used DDR1 (D1G6, Cell Signaling Technology Inc. catalogue #5583) rabbit mAb antibody. Loading controls β actin (8H10D10) Mouse mAb (#3700) and alpha tubulin antibody (#2148) were all from Cell Signaling Technology Inc. For apoptosis and downstream EGFR signaling, cells were treated with 3uM Gefitinib for 24 hours and treated with 24nM Ros1 inhibitor for 12 hours. Antibody for p44/p42 MAPK (Erk1/Erk2) is for dually phosphorylated Erk1 (T202, Y204) and Erk2 (T185, Y187) or singly phosphorylated at T202. We only picked up Erk2 (T185, Y187). All antibodies are from Cell Signaling Technologies: p44/p42 MAPK catalogue #4370; PARP catalogue #9542; pAKT catalogue #2965 and Beta Actin catalogue #3700.

### Flow cytometry for cell cycle profiling

For cell cycle analysis, cell staining with propidium iodide (PI) was used to determine DNA content following the standard protocol. The profiles were obtained using Beckman Coulter™ Cell Lab Quanta flow cytometer. Data was analyzed using Cell Lab Quanta SC and Modfit Lt™ DNA data analysis software.

## SUPPLEMENTARY FIGURES


