# Supplementary material for: ROS1 amplification mediates resistance to gefitinib in glioblastoma cells
Source: Oncotarget. 2015 May 4;6(24):20388–95. doi: 10.18632/oncotarget.3981 (PMC4653012; doi:10.18632/oncotarget.3981)
Supplement: Supplementary file 1 [file oncotarget-06-20388-s001.pdf]

# ROS1 amplification mediates resistance to gefitinib in glioblastoma cells

**Supplementary Material**

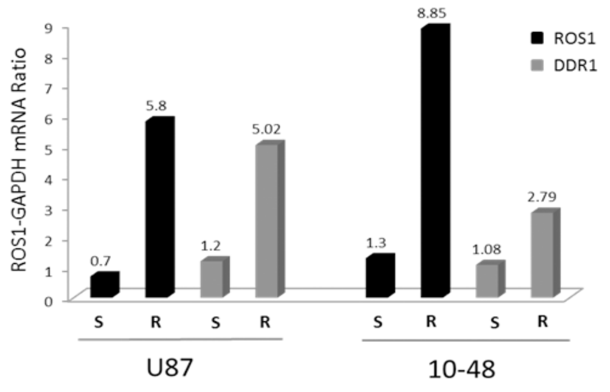


**Fig. S1: Activation of ROS1 and DDR1 in low passage tumor-derived GMB 10-48 cell line.** The 10-48 GBM cell lines (passage 7) was treated with increasing concentrations of gefitinib similar to what was described for U87 cells. The resistant clones were pooled and tested by real time PCR for the transcript levels of ROS1 and DDR1. Pooled U87 resistant cells were used as a control. S indicates sensitive cells and R indicates resistant cells.

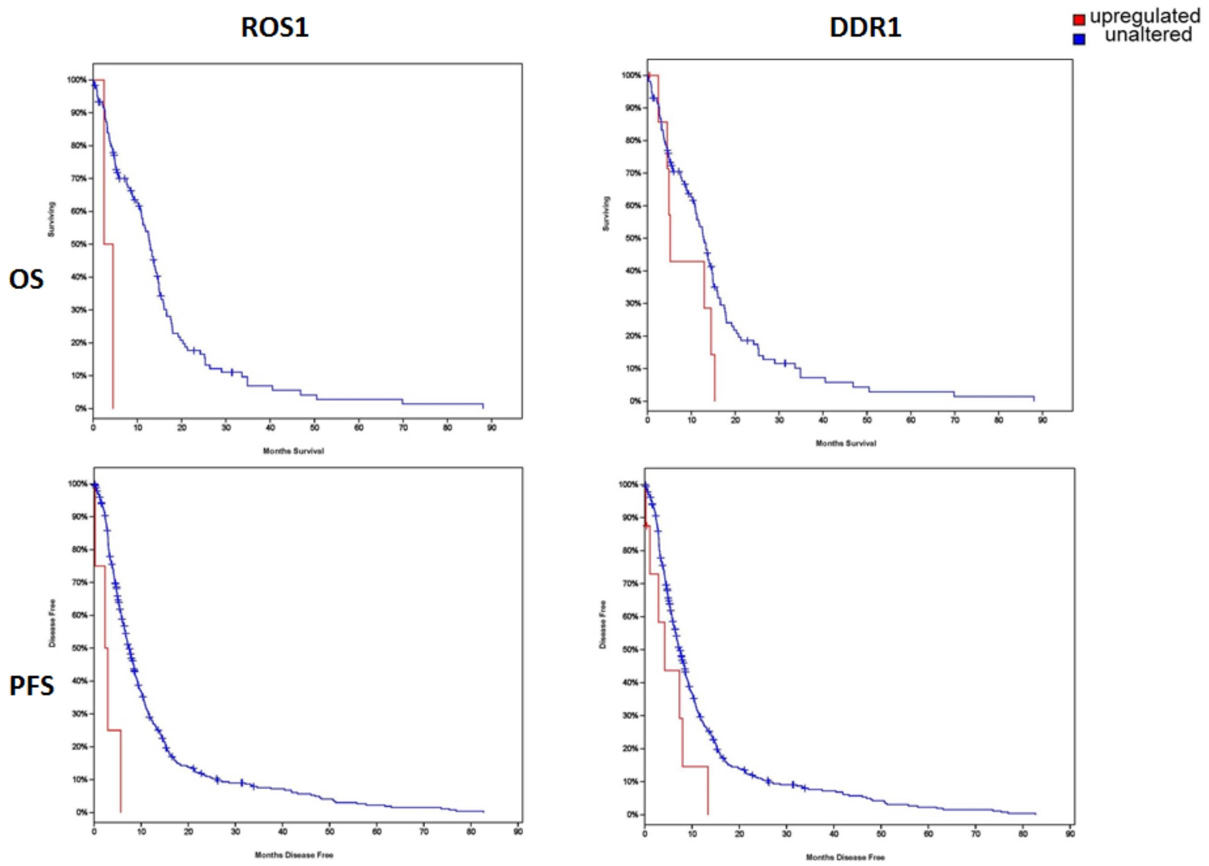


**Fig. S2. Overall survival and disease free survival in GBM patients with upregulated ROS1 and DDR1 proteins.** Survival data from TCGA indicates that GBM patients with an upregulated ROS1 or DDR1 have a worst survival outcome.

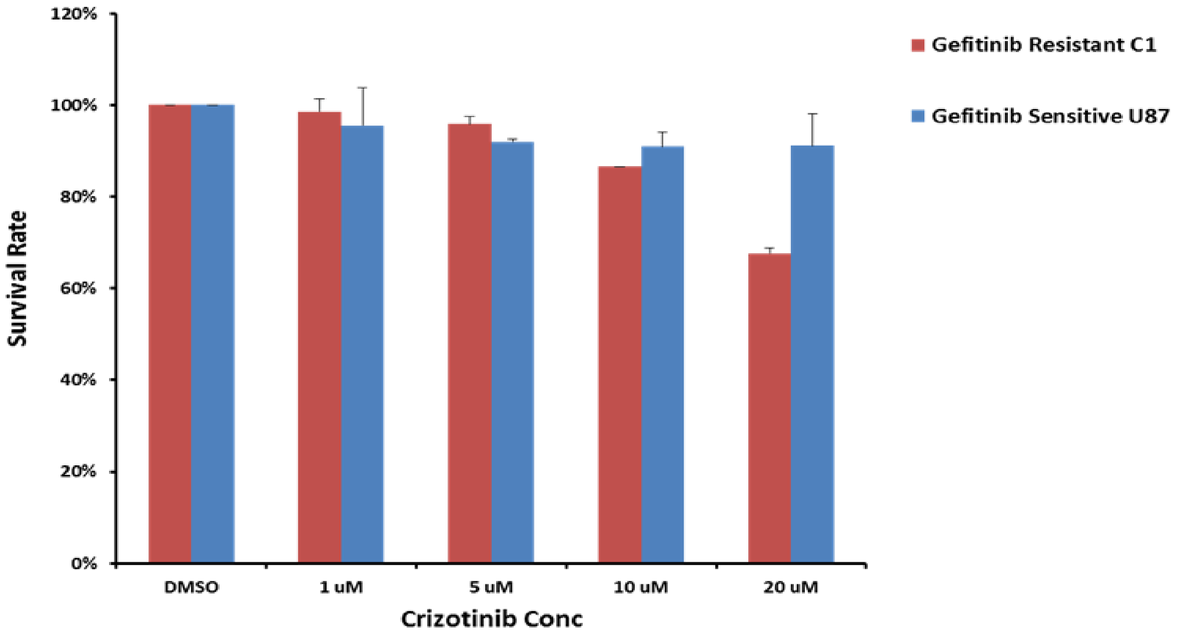


**Fig. S3. Treatment of gefitinib-resistant cells with crizotinib.** The gefitinib-resistnt clone C1 was treated with increasing concentrations of crizotinib. While cells show some sensitivity at higher concentrations of the drug, they remain less sensitive than to the pyrazole ROS1 inhibitor.
